# Mosaic Labeling and 3-Dimensional Morphological Analysis of Single Cells in the Zebrafish Left-right Organizer

**DOI:** 10.21769/BioProtoc.3090

**Published:** 2018-11-20

**Authors:** Agnik Dasgupta, Andrew E. Jacob, Jeffrey D. Amack

**Affiliations:** 1 Department of Cell and Developmental Biology, State University of New York Upstate Medical University, Syracuse, NY, USA;; $a Current address: Laboratory of Sensory Neuroscience, Rockefeller University, New York City, USA;; $b Current address: Department of Embryology, Carnegie Institution of Washington, Baltimore, MD, USA

**Keywords:** Left-right organizer, Dorsal forerunner cells, Kupffer’s vesicle, Mosaic labeling, Zebrafish embryo, 3D reconstructions, Single cell, Morphometric analysis

## Abstract

A transient epithelial structure called the left-right organizer (LRO) establishes left-right asymmetry in vertebrate embryos. Developmental defects that alter LRO formation result in left-right patterning errors that often lead to congenital heart malformations. However, little is known about mechanisms that regulate individual cell behaviors during LRO formation. To address this, we developed a Cre-*loxP* based method to mosaically label precursor cells, called dorsal forerunner cells, that give rise to the zebrafish LRO known as Kupffer’s vesicle. This methodology allows lineage tracing, 3-dimensional (3D) reconstruction and morphometric analysis of single LRO cells in living embryos. The ability to visualize and quantify individual LRO cell dynamics provides an opportunity to advance our understanding of LRO development, and in a broader sense, investigate the interplay between intrinsic biochemical mechanisms and extrinsic mechanical forces that drive morphogenesis of epithelial tissues.

## Background

Recent efforts in the field of developmental biology have focused on understanding mechanisms underlying tissue and organ morphogenesis at the single-cell level. Taking advantage of the transparent zebrafish embryo–which is a useful system for conducting *in vivo* cell biology experiments–several approaches have been developed to analyze the dynamics of single cells in living embryos. Transient expression of injected mRNAs or transgene constructs has been widely used to mosaically label cells with fluorescent proteins for analysis of individual cells in complex environments, such as endothelial cells in the developing vasculature (Yu *et al*., 2015). Optogenetic approaches have used light to change the fluorescent properties of photo-convertible proteins (Schuster and Ghysen, 2013) or uncage fluorescent dextran (Clanton *et al*., 2011) in single cells. Taking a genetic approach, a transgene that contains three fluorescent proteins–RFP (red), YFP (yellow), and CFP (cerulean)- separated by recombination (*lox*) sites that was first used to engineer ‘brainbow’ mice (Livet *et al*., 2007) was used to generate stable ‘Zebrabow’ transgenic zebrafish (Pan *et al*., 2013). Expression of Cre recombinase in Zebrabow embryos generates differential fluorescent labeling of individual cells based on the stochastic recombination of the Zebrabow transgene. Each of the approaches mentioned here have been used successfully to analyze single cells during development and/or regeneration processes in zebrafish.

**Figure 1.**
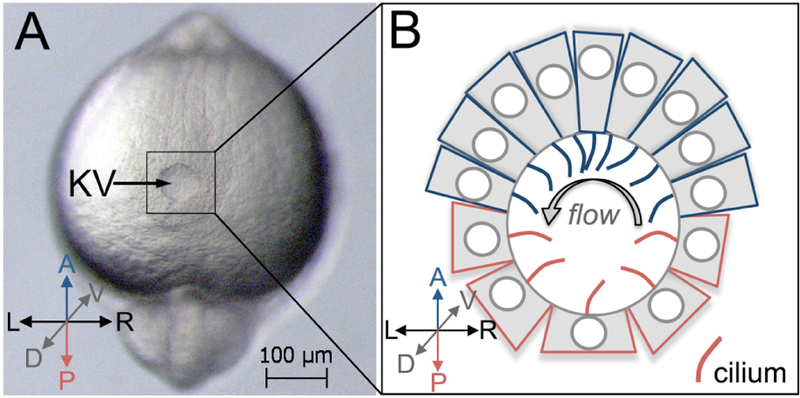
Kupffer’s vesicle in the zebrafish embryo. A. A dorsal view of Kupffer’s vesicle (KV) in a live zebrafish embryo at 8-somite stage (8 ss) of development. This is a brightfield image taken using a Zeiss Discovery V12 stereomicroscope. B. A schematic diagram of KV shows cell shapes at middle focal plane, and cilia (red and blue) projecting into the lumen to drive fluid flow within the KV after remodeling at 8 ss. A = Anterior; P = Posterior, L = Left; R = Right; D = Dorsal; V = Ventral. Anterior KV cells are represented in blue and posterior KV cells are red. Arrow = strong leftward flow.

**Figure 2.**
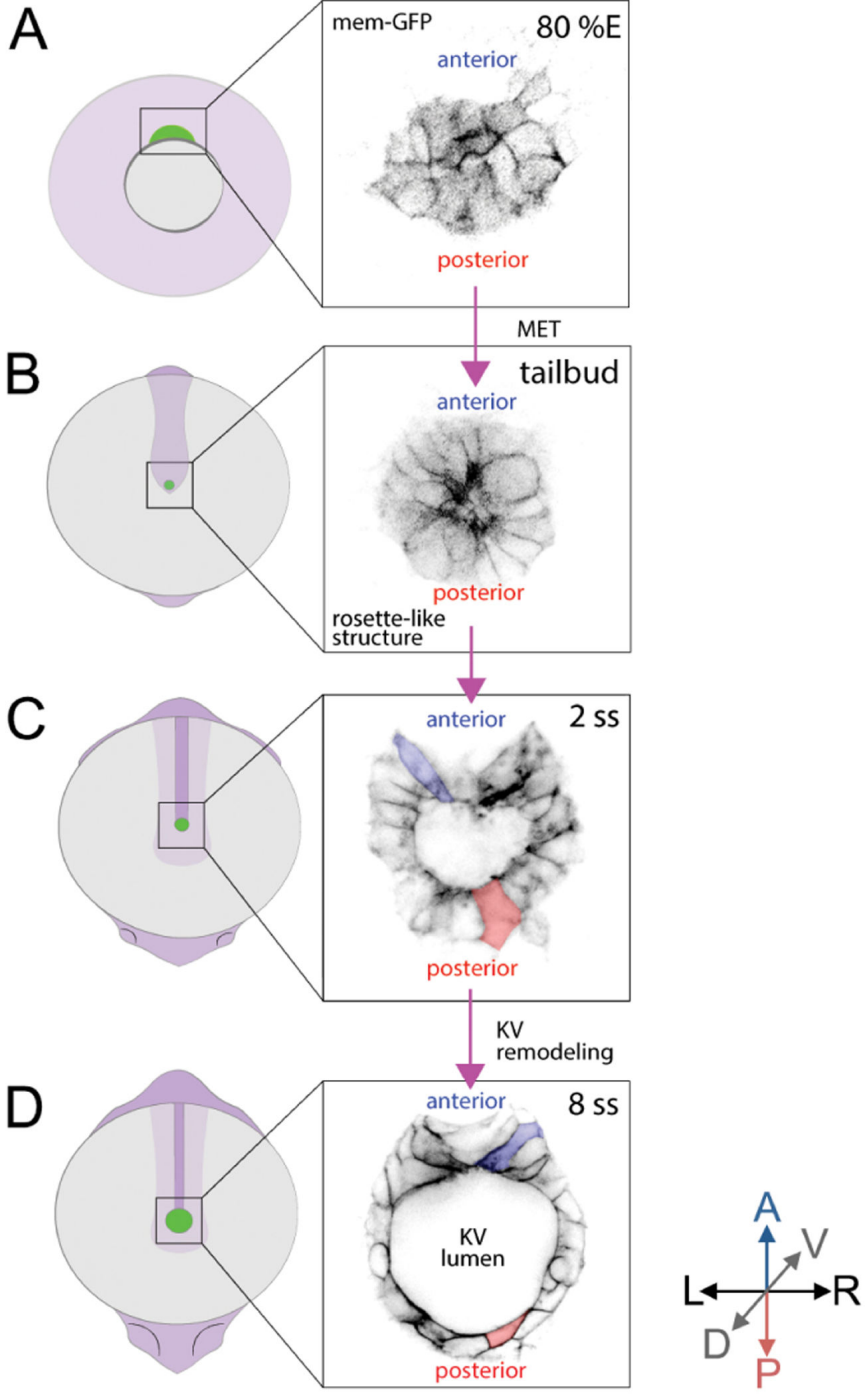
Behavior and 2D morphology of DFC and KV cells during development. Two transgenic zebrafish strains–*Tg(dusp6:memGFP)* and *Tg(sox17:GFP-CAAX)*–provide bright labeling of dorsal forerunner cells (DFCs) that give rise to Kupffer’s vesicle (KV) cells. A and B. Embryo diagrams (green represents DFC/KV cells) and inversed fluorescence images of membrane-localized GFP expression in DFCs in *Tg(dusp6:memGFP)* transgenic zebrafish at 80% epiboly stage (80% E) (A) and the tailbud stage (B) when migratory DFCs form a rosette structure. C and D. GFP expression in *Tg(sox17:GFP-CAAX)* transgenic zebrafish marks KV cell membranes during KV lumen formation at the 2 somite stage (2 ss) (C) and in the mature organ at 8 ss (D) after KV remodeling. A = Anterior; P = Posterior, L = Left; R = Right; D = Dorsal; V = Ventral. Anterior cells = blue, Posterior cells = red.

Kupffer’s vesicle (KV) (Figure 1A) functions as a left-right organizer (LRO) in the zebrafish embryo (Essner *et al*., 2005; Kramer-Zucker *et al*., 2005) that is analogous to the ventral node in mouse and the gastrocoel roof plate in frog (Blum *et al*., 2014). Motile cilia in the LRO (Figure 1B) generate asymmetric signals to establish the left-right body axis in developing vertebrate embryos, which is critical for normal development of the cardiovascular and gastrointestinal systems (Dasgupta and Amack, 2016; Grimes and Burdine, 2017). Fate-mapping studies have identified the precursor cells–called dorsal forerunner cells (DFCs)–that give rise to the zebrafish LRO (KV) (Cooper and D’Amico, 1996; Melby *et al*., 1996). Multiple transgenic tools, including *Tg(sox17:GFP-CAAX)* (Dasgupta *et al*., 2018) and *Tg(dusp6:memGFP)* (Wang *et al*., 2011), have been developed to visualize and quantify the behaviors of DFCs and KV cells. DFCs migrate as mesenchymal cells during gastrulation stages (Figure 2A), undergo a mesenchymal-to-epithelial transition (MET) at the end of gastrulation, and then form a rosette-like structure (Amack *et al*., 2007; Oteiza *et al*., 2008) (Figure 2B). Next, the KV lumen expands, and each KV cell extends a motile cilium into the fluid-filled lumen. During KV lumen expansion, epithelial KV cells at the middle plane of the organ that start out with similar morphologies (Figure 2C) undergo a morphogenetic process that we refer to as ‘KV remodeling’. During this process, cells in the anterior region of KV increase in size and develop columnar shapes that allow tight packing of these cells. In the posterior region of KV, cells decrease in size and become wide and thin (Figure 2D). KV remodeling creates an asymmetric distribution of motile cilia along the anteroposterior axis–with more cilia packed into the anterior region (Figure 1B)–that is necessary to generate right-to-left asymmetric fluid flows in KV and left-right patterning in the embryo (Wang *et al*., 2011 and 2012). Probing KV development at the single-cell level will be essential to understanding the relationship between intrinsic and extrinsic mechanisms that mediate asymmetric epithelial morphogenesis in KV.

**Figure 3.**
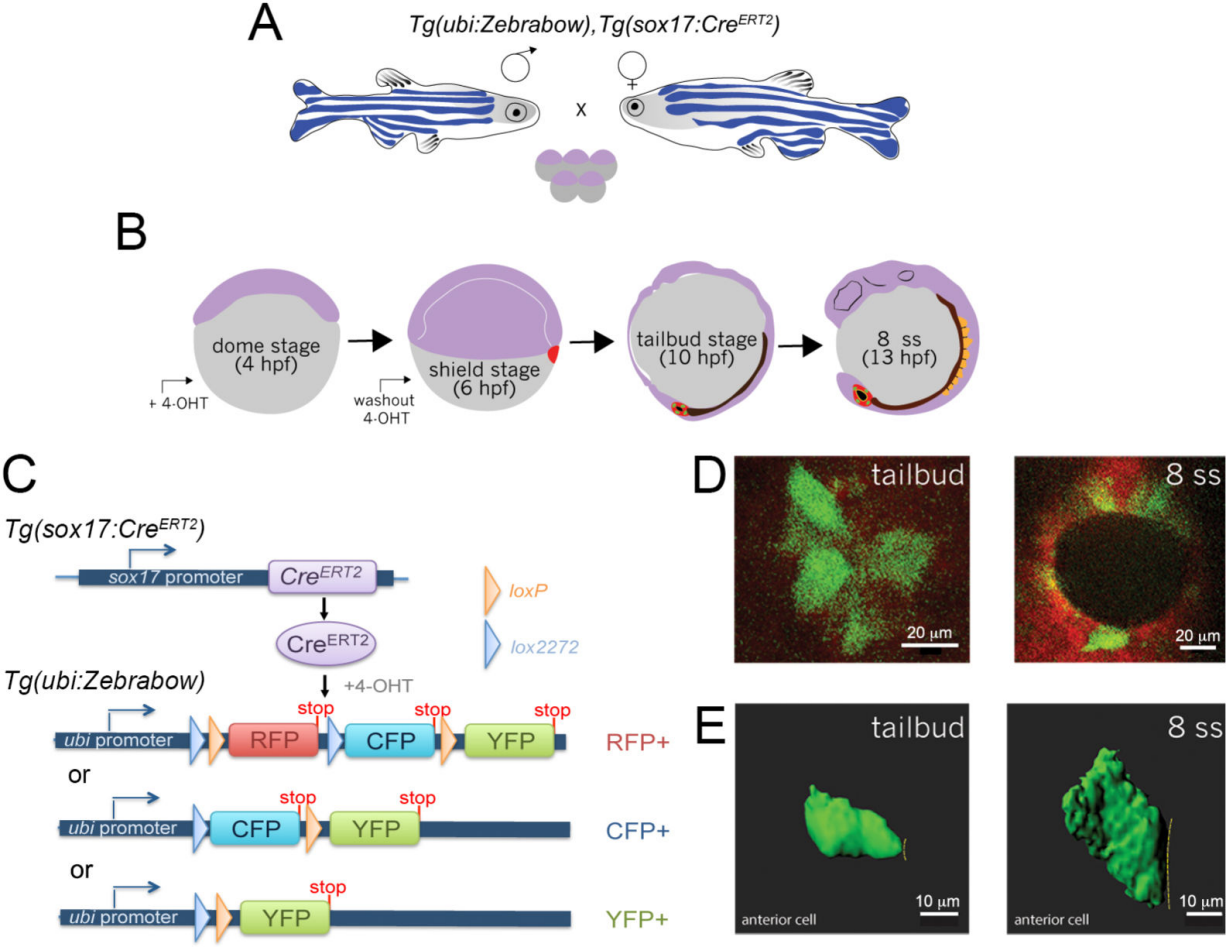
Mosaic labeling and 3D rendering of single KV cells. A. Double transgenic *Tg(sox17:Cre*^*ERT2*^*); Tg(ubi:Zebrabow)* zebrafish are incrossed to obtain embryos. B. Time course of mosaic labeling of KV cells. Brief treatment of double transgenic *Tg(sox17:Cre*^*ERT2*^*); Tg(ubi:Zebrabow)* embryos with 4-OHT from the dome stage (4 h post-fertilization) to the shield stage (6 hpf) generates low levels of Cre activity that changes expression of default RFP to expression of CFP or YFP in a subset of KV cells. C. Structure of the *ubi:zebrabow* and *sox17:Cre*^*ERT2*^ transgenes and the possible recombination outcomes of the Zebrabow transgene by Cre recombinase activity in KV cell lineages. Cre can mediate the deletion of sequences flanked by *loxP* sites (orange triangles) or variant *lox2272* sites (blue triangles), leaving behind single *loxP* or *lox2272* sites that are not cross-compatible with each other. D. Mosaic labeled YFP^+^ KV cells (pseudo-colored green) at the middle plane of KV at tailbud stage and 8 somite stage (8 ss). Scale bars = 20 μm. E. 3D reconstructions of single KV cells (green) using Imaris software at tailbud and 8 ss. Dashed line indicates KV lumen surface. Scale bars = 10 μm.

Herein we describe a genetic mosaic labeling strategy to fluorescently label individual KV cells and provide a guide to analyze 3D data obtained from imaging live mosaic-labeled embryos using Imaris software. We have generated stable transgenic *Tg(sox17:Cre*^*ERT2*^*)*^*sny120*^ zebrafish, in which a *sox17* promoter drives the expression of a tamoxifen-inducible Cre recombinase (Cre^***ERT2***^) (Feil *et al*., 1997) in the DFC/KV cell lineage and endodermal cells. To take advantage of Cre-*loxP* based cell labeling in Zebrabow embryos, we created double transgenic fish to express the *Tg(sox17:Cre*^*ERT2*^*)* transgene in a *Tg(ubi:Zebrabow-M)*^*a131*^ background (Pan *et al*., 2013) in which the zebrafish ubiquitin (ubi) promoter drives the expression of the Zebrabow transgene in all cells (Figure 3A). We next determined a dose of 4-hydroxytamoxifen (4-OHT) that induces low levels of Cre activity in DFCs, and reliably results in mosaic labeling of DFC/KV cells in *Tg(sox17:Cre*^*ERT2*^*); Tg(ubi:Zebrabow)* embryos (Figure 3B). The low Cre activity switches default RFP expression to CFP or YFP expression in a subset of cells (Figures 3C and3D). Confocal images of single mosaic-labeled cells in live embryos can be used to reconstruct and quantify 3D cellular morphology (Figure 3E). This approach provides a simple and efficient method to stochastically label individual DFC/KV cells for analysis of cell behaviors in real-time during morphogenesis of the zebrafish LRO.

## Materials and Reagents

Petri dish (VWR, catalog number: 25384–088)
12-well clear flat bottom not treated multiwell cell culture plate (Falcon, catalog number: 351143)
Glass bottom microwell dishes, 35 mm Petri dish, 14 mm microwell, No. 1.5 coverglass (0.16–0.19 mm) (MatTek, catalog number: P35G-1.5–14-C)
Glass transfer pipet (Fisher, catalog number: 63A183–624)
Double transgenic *Tg(sox17:Cre*^*ERT2*^*); Tg(ubi:Zebrabow)* zebrafish (available upon request from the Amack lab: amackj@upstate.edu)
  Note: The Tg(sox17:Cre^ERT2^); Tg(ubi:Zebrabow) strain is maintained by selecting embryos to raise that have green fluorescent hearts (cmlc2:GFP expression is a marker for the sox17: Cre^ERT2^ transgene) and bright ubiquitous RFP expression from the ubi:Zebrabow transgene.
(Z)-4-Hydroxytamoxifen (4-OHT) (Sigma, catalog number: H7904)
  Note: Reconstituted to a stock solution of 10 mM in 1% DMSO and stored at −20 °C in single-use aliquots.
1% low-melting point (LMP) agarose (Invitrogen, catalog number: 15517–014) prepared in embryo medium and maintained at 50 °C
1% agarose (VWR, catalog number: 0710–500G) prepared in embryo medium that will be used to coat the bottom of Petri dishes and 12-well plates
Dimethyl sulfoxide (DMSO) (VWR, catalog number: 0231–500mL)
Sodium chloride (NaCl) (Fisher, catalog number: BP358–212)
Potassium chloride (KCl) (J. T. Baker, catalog number: 3040–01)
Calcium chloride dihydrate (CaCl_***2***_·2H_***2***_O) (J. T. Baker, catalog number: 1332–01)
Magnesium sulfate heptahydrate (MgSO_***4***_·7H_***2***_O) (Fisher, catalog number: BP214–500)
Methylene blue (Fisher, catalog number: BP117–100)
Embryo medium (see Recipes)

## Equipment

1. Tweezers (Electron Microscopy Sciences, catalog number: 0103–5-PS)
  Note: We use Dumont Tweezer style 5.
2. Incubator set at 28.5 °C for culturing zebrafish embryos (VWR, catalog number: 35960–056)
3. Stereomicroscope with epifluorescent light source (Zeiss, model: Stereo Discovery V12)
  Note: We use a Zeiss Stereo Discovery V12.
4. Confocal microscope (Nikon, model: Eclipse Ti)
  Note: We use a Perkin-Elmer UltraVIEW Vox spinning disc confocal system equipped with 488 nm and 561 nm solid-state lasers (for YFP and RFP excitation) mounted on a Nikon Eclipse Ti inverted microscope with a Hamamatsu C9100–50 EM-CCD camera. We use a 20× oil-immersion objective. The microscope is equipped with a temperature control chamber for live imaging. It is likely that this protocol can be adapted for use with a laser scanning confocal microscope, but we have not tested different microscope platforms.
5. Freezer

## Software

1. Volocity (PerkinElmer) for image acquisition
2. Imaris (BitPlane) for 3D rendering and morphometric analysis

## Procedure

**Figure 4.**
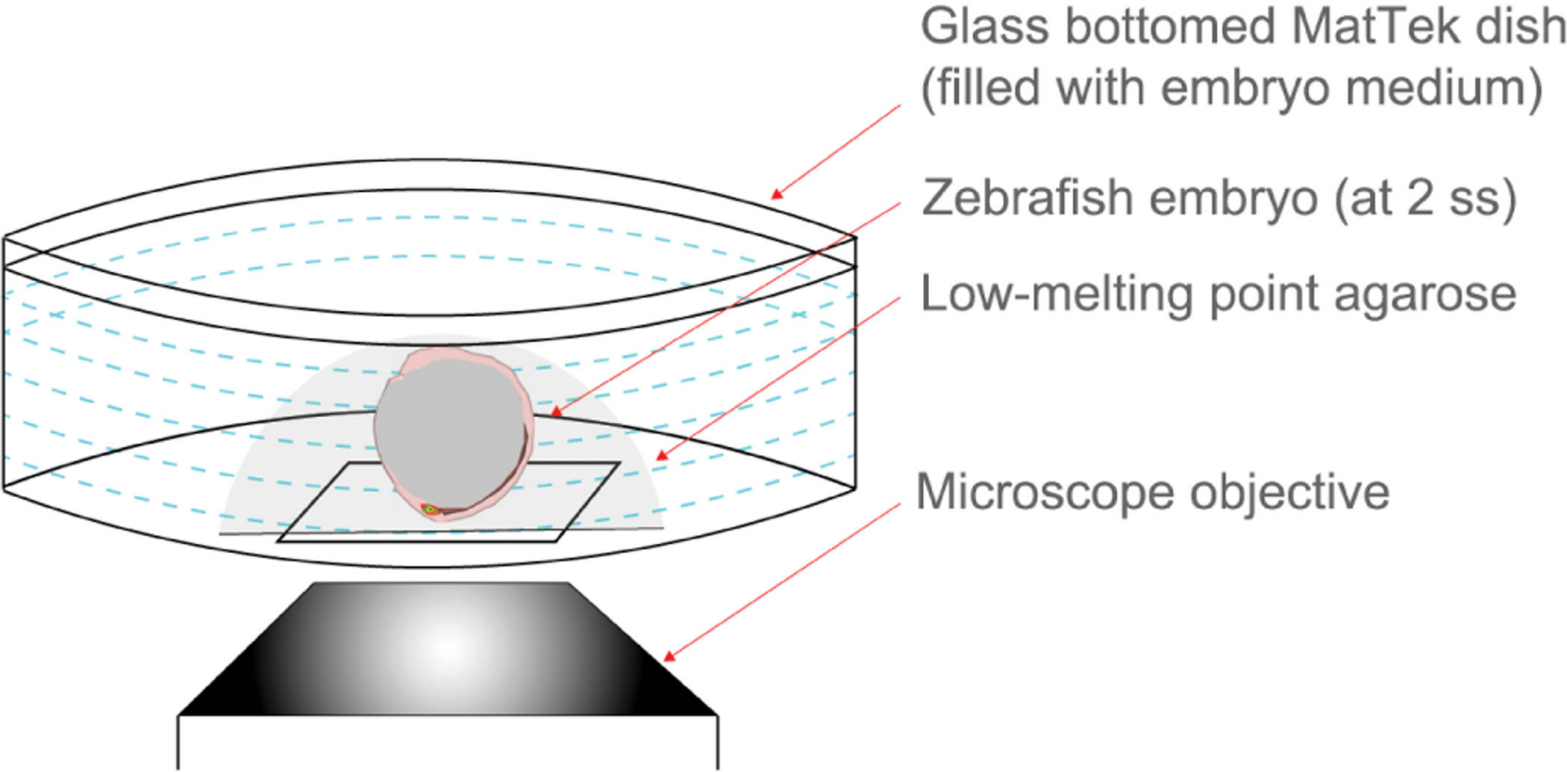
Immobilization of mosaic labeled embryos for live imaging. Schematic representing embryo immobilization technique used for live imaging with an inverted confocal microscope. A live embryo is covered with liquid low-melting point (LMP) agarose in a MatTek dish and then positioned such that DFC/KV cells are close to the glass bottom. Once solidified, the agarose is covered by embryo medium.

- Fluorescent mosaic labeling of DFC/KV cells
  1. Set up crosses of homozygous double transgenic *Tg(sox17:Cre*^*ERT2*^*); Tg(ubi:Zebrabow)* zebrafish in breeding tanks with dividers that separate males from females. Remove dividers at the desired time to allow fish to breed and synchronize embryo development.
  2. Collect *Tg(sox17:Cre*^*ERT2*^*); Tg(ubi:Zebrabow)* embryos and culture them in embryo medium in a Petri dish at 28.5 °C until they reach the dome stage of development ~4 h post-fertilization (hpf).
  3. Carefully remove embryos from their chorion using fine tweezers (we use Dumont Tweezer style 5 from Electron Microscopy Sciences) in a Petri dish coated with 1% agarose. The agarose prevents the yolk of embryo from sticking to the plastic surface of Petri dish. To coat the dish, pipet enough hot liquid 1% agarose to cover the bottom of the dish, and then allow it to cool and solidify.
  4. Transfer dechorionated embryos using a glass transfer pipet (fire polish the tip) to a 12-well flat bottom cell culture plate coated with 1% agarose. Replace the embryo medium with fresh embryo medium containing 5 μM 4-hydroxytamoxifen (abbreviated here as 4-OHT) and 0.1% DMSO (dimethyl sulfoxide). The DMSO aids in cell permeability and drug delivery. Treat control embryos with 0.1% DMSO alone. We recommend that each well contain 5–6 dechorionated embryos.
  5. Incubate embryos in 5 μM 4-OHT medium from the dome stage (4 hpf) to the shield stage (6 hpf) at 28.5 °C.
  6. At the shield stage, transfer treated embryos to fresh embryo medium without 4-OHT and gently swirl. Repeat this step 3 times with fresh embryo medium to wash out 4-OHT.
  7. Return rinsed embryos to 28.5 °C to allow development to the desired stage for imaging labeled DFC/KV cells.
    Note: Results from our work (Dasgupta et al., 2018) indicate Cre activity is not spatially biased, but randomly labels cells throughout the KV. In addition, we found on average that imaging 12 embryos will result in ~20 anterior KV cells and ~20 posterior KV cells to analyze.
- Immobilization of mosaic labeled embryos for imaging using an inverted microscope
  1. At the desired stage of development, carefully transfer an embryo to a glass-bottom (MatTek) dish using a glass transfer pipet. To analyze DFC behaviors, embryos can be prepared at any stage during epiboly. To visualize KV morphogenesis, we prepare embryos between the 1–2 somite stages.
    Note: Accumulation of YFP expression is time-dependent following Cre activation (4-OHT treatments). Thus, YFP fluorescence is weak at early (epiboly) stages and brighter at later (somite) stages.
  2. After transferring the embryo to the MatTek dish, remove most of the embryo medium and then cover the embryo with liquid 1% low-melting point (LMP) agarose that was maintained at 50 °C.
  3. While the agarose solidifies, use a stereomicroscope to orient the embryo so that the DFC/KV cells face the glass-bottom (Figure 4; Video 1).
  4. Once the agarose has solidified, and the embryo is immobilized, add embryo medium to the dish to cover the sample and prevent the sample from drying out.
    Note: It is recommended to repeat this process to mount 5+ embryos for screening to identify the embryo(s) with the degree of mosaic labeling that is appropriate for the designed experiment.
- Imaging mosaic labeled DFC/KV cells in live embryos
  1. Position the MatTek dish containing the immobilized live embryo on an inverted confocal microscope. We use a 20× objective on a Perkin-Elmer UltraVIEW Vox spinning confocal disc confocal system with an environmental chamber maintained at 32 °C to image live embryos. *Note: The Tg(ubi:Zebrabow) transgene drives RFP expression in all cells by default, which we excite using a 561 nm laser. If Cre-mediated recombination has occurred in a cell, we observe YFP expression (excited with a 488 nm laser)*.
  2. Select an embryo with bright YFP^+^ mosaic labeling that allows individual cells to be distinguished from their neighbors (Figure 3D). Laser power and exposure time will depend on signal intensity. Typically, we use 488 nm laser power between 30% and 50%, and exposure times between 500 ms and 800 ms using the 20× objective. Power for the 561 nm laser is typically 30% with an exposure time of 100–300 ms.
    Note: Laser power and exposure time should be minimized to prevent photo-toxicity. To achieve this, we suggest selecting mosaic labeled embryos with the brightest YFP expression.
  3. To analyze a single time point, we capture a Z-series through the entire KV using 2 μm Z steps. The typical distance is ~35 Z steps (70 μm) at the 2 somite stage and ~45 Z steps (90 μm) at the 8 somite stage.
  4. For time-lapse imaging, we capture Z-stacks through the entire KV every 5 min during KV morphogenesis.
    Note: We have imaged a single embryo (using 5 min intervals) for up to 3 h (between 2 ss and 8 ss) without detecting photo-damage or deleterious effects on embryo development.
- Viewing confocal images using Imaris software
  1. To open a confocal image (Z-stack) in Imaris software, the raw data will need to be converted to an OME TIFF (**.ome**) file. In Volocity software, select the image to convert, and then right-click to export the file. Save the file as an OME TIFF.
  2. In Imaris (we have used version 8.4.0), click the **Assay** icon to create a new project folder. Next, click the **Group** icon to create a new group within the assay. Finally, click the **Image** icon and use the open file window to add an image (Z-stack in .ome file format) to the group.
  3. Double click on the .ome file icon to open the image in Imaris. This will open in the **Surpass** view.
  4. Next, open the **Image Properties** window (CTRL-I). Set the desired color for each channel (*e.g*., green, red, blue, *etc*.).
  5. Open the **Geometry** tab within the **Image Properties** window. Set the voxel size using pixel dimensions and Z-step size used to capture the image. Pixel dimensions are measured manually for each objective using a micrometer. For example:

**Table:** 

Spinning disc confocal 20× objective:	X = 0.33 (1 pixel = 0.33 μm)
	Y = 0.33
	Z = step size (2 μm) used to acquire Z-stack
  6. Open the **Adjustment** window (CTRL-D) to adjust signal levels.
  7. Click **Store** to save the processed image.
- 3D rendering of mosaic labeled DFC/KV cells using Imaris software
  1. To 3D render an object (*e.g*., single cell), click **Surface** under the 3D view menu.
  2. To define the region of interest (ROI) for rendering, go to the **create** tab and check **Segment only a region of interest** box.
  3. Click the **next** (blue) button at the bottom of the menu sidebar. The ROI bounding box appears (Figure 5A).
  4. To change the size of the bounding box, the cursor must be in the **Select** mode. Use the **ESC** key to toggle between **Navigate** and **Select** modes.
  5. In **Select** mode, click and drag arrowheads to re-size the bounding box in X, Y and Z around a single cell that you would like to reconstruct (Figure 5B).
    Note: you cannot zoom or rotate in Select mode; you must toggle to Navigate mode.
  6. Click **next**.
  7. Select **source signal** (*e.g*., green channel that includes YFP^***+***^ cells).
  8. Set **surface detail**. A higher number is more smooth and less detail (*e.g*., 1 = smooth).
  9. Set **thresholding**. Fifteen micrometer works well for KV cells. Smaller background signals are ignored.
    Note: The thresholding number should be based on the size of the object (cell) that you are rendering. The length and width of the cell can be measured manually in the 2D slice view.
  10. Click **next**.
  11. The slider can be used to manually adjust level of 3D rendering of the cell. Use navigate mode to check the rendering in X, Y, and Z.
  12. Optional: If a cell of interest is in contact with another labeled cell, use **Split Touching Objects** function. Check **Enable** and set the **Seed Point Diameter**. A value of 8 is often good for KV cells.
  13. Click **next**.
  14. Dots show how many labeled cells the software detects.
  15. If necessary, go back and change **Seed Point Diameter** until the number of cells is accurate.
  16. Click the **next** button to finish. This completes the 3D rendering a single KV cell completes (Figure 5C).
  17. To edit a 3D rendering, select **edit** (pencil icon in the lower menu bar).
  18. Select the cell you wish to analyze and click **duplicate** in the **edit** tab. This creates a new surface file with only the selected 3D rendered cell.
  19. To obtain measurements of the 3D rendering, click **statistics** (graph icon in the lower menu bar), select **detailed** tab, select **specific values** and use the drop-down menu to select a measurement (*e.g*., area, volume, intensity, *etc*.).
  20. Use **clipping plane** (scissors icon) to slice through the 3D rendered image. This tool can be used to slice 3D surface rendered KV cells to measure cell cross-sectional areas.
  21. Use **snapshot** to capture an image (3D rendering, cross-section, *etc*.). Under the file tab, make a copy and save.
  22. Representative data are shown in Figure 6.

## Data analysis

Taking measurements of 3D rendered DFC/KV cells using Imaris software.

1. Click **new measurement points** icon in the top menu bar with small icons.
2. In the settings tab, select **sphere** (check the box).
3. For **line mode** check **pairs**.
4. Select **edit** tab.
5. Under **intersect with**, select **surface of an object**.
6. In **Select** mode, a box appears at the cursor.
7. Hold down the **shift** key and click to mark the first point of the line.
8. Repeat to mark the second point of the line.
9. To adjust the line, select one point (turns yellow), hold down shift and drag to new location.
10. Use **settings** tab to change font or color of the line.
11. Click **statistics** icon to obtain measurements in the **detailed** tab. Results can be saved as an excel file by clicking the **export statistics** (floppy disc icon) at the bottom of the menu sidebar.
12. Use **snapshot** to capture an image. Under the file tab, make a copy and save.

## Notes

**Figure 5.**
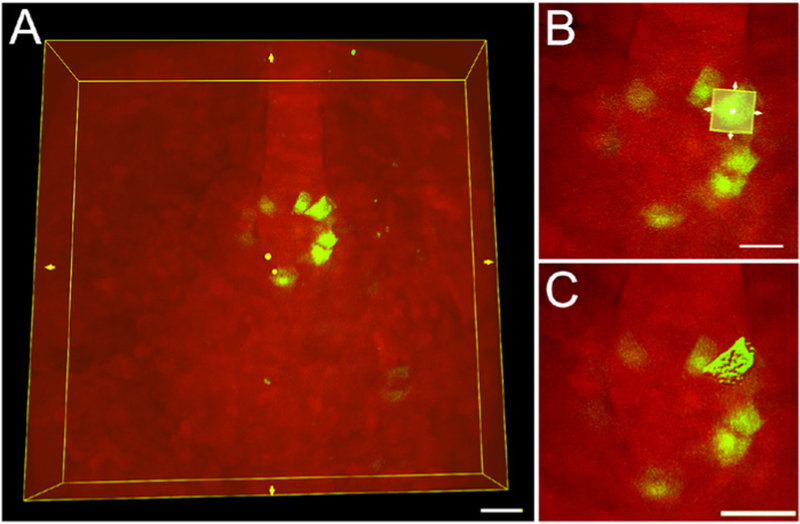
3D rendering of a single cell using a region of interest in Imaris software. A. A region of interest (ROI) bounding box (yellow box) around an entire 3D image of mosaic-labeled KV cells. Scale bar = 30 μm. B. The ROI bounding box can be re-sized to include only a single cell. Scale bar = 20 μm. C. The software 3D renders only the cell included in the ROI. Scale bar = 30 μm.

We realized Imaris is expensive software that is not available at all institutions. Alternative software packages such as Volocity (Perkin Elmer) or FIJI/ImageJ (free download at https://imagej.net/Fiji/Downloads) can be used to visualize confocal data sets in 3D, and generate 3D reconstructions for analysis. We focus this protocol on using Imaris because of its capability to segment a region of interest and 3D reconstruct a single cell (Figure 5).

## Recipes

1. Embryo medium

**Table:** 

Reagent	Quantity	Final concentration
NaCI	2.94 g	5.03 mM
KCI	0.13 g	0.17 mM
CaCI_2_·2H_2_O	0.49 g	0.33 mM
MgSO_4_·7H_2_O	0.81 g	0.33 mM
Methylene blue	10 g	0.1% (w/v)
Purified water (reverse osmosis)	10 L	Not applicable

## Supplementary Material

Video 1 **Video 1. Immobilization of a live embryo for confocal microscopy**. This video demonstrates Steps 1–4 of Procedure B.

## Figures and Tables

**Figure 6.**
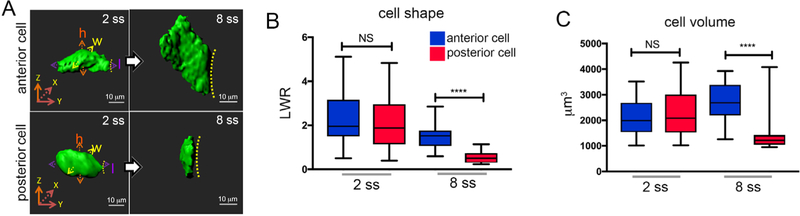
Quantification of 3D rendered KV cells using Imaris. A. 3D renderings of cells in the anterior or posterior region of KV at the 2 somite stage (2 ss) or 8 somite stage (8 ss). Cell height (h), length (l), and width (w) measurements are shown at 2 ss. Dashed line indicates KV lumen surface. Scale bars = 10 μm. B-C. Box and whisker plots showing quantification of length to width ratio (LWR) that describes the shape of KV anterior or posterior cells (B), and quantification of cell volumes (C). n = 27 anterior cells at 2 ss; n = 25 posterior cells at 2 ss; n = 21 anterior cells at 8 ss; n = 22 posterior cells at 8 ss. Anterior and posterior KV cells have similar shapes and volumes at 2 ss, and then undergo asymmetric morphological changes that result in different cell shapes and volumes at 8 ss. n = number of cells analyzed. NS = not significant; ****P < 0.001. These images and results are modified from Dasgupta *et al*. (2018).
